# A prognostic framework integrating endoplasmic reticulum stress dynamics reveals clinical stratification and differential prognostic attributes in osteosarcoma patients

**DOI:** 10.3389/fmed.2025.1566387

**Published:** 2025-10-01

**Authors:** Yan Wang, Sina Ahmadi, Mingchao Ding, Yinggang Che, Changlong Song, Sijia Na, Weiqi Wang

**Affiliations:** ^1^State Key Laboratory of Oral and Maxillofacial Reconstruction and Regeneration, National Clinical Research Center for Oral Diseases, Shaanxi Clinical Research Center for Oral Diseases, Department of Oral and Maxillofacial Surgery, School of Stomatology, The Fourth Military Medical University, Xi’an, China; ^2^Key Laboratory of Shaanxi Province for Craniofacial Precision Medicine Research, Department of Shaanxi, Oral and Maxillofacial Surgery, College of Stomatology, Xi'an Jiaotong University, Xi’an, China; ^3^Department of Respiratory Medicine, Air Force Hospital of Western Theater Command, PLA, Chengdu, China

**Keywords:** endoplasmic reticulum stress, osteosarcoma, prognostic model, immune traits, IL4

## Abstract

**Background:**

Endoplasmic reticulum (ER) stress is recognized as a pivotal factor in the initiation and advancement of osteosarcoma; however, its implications for patient prognosis remain poorly understood.

**Methods:**

Our objective was to elucidate the prognostic implications and immune infiltration patterns associated with endoplasmic reticulum (ER) stress in osteosarcoma patients through the synthesis of existing osteosarcoma datasets and the application of advanced bioinformatics methodologies.

**Results:**

Our findings elucidate distinct and heterogeneous expression patterns of endoplasmic reticulum (ER) stress-related genes in osteosarcoma, contrasting sharply with those identified in osteocytes and mesenchymal stem cells. We developed a robust ER stress model comprising ten ER stress-associated genes specifically tailored for osteosarcoma patients. This model was constructed utilizing univariate analysis and least absolute shrinkage and selection operator (LASSO) regression techniques. The predictive robustness and applicability of the model were ascertained through receiver operating characteristic (ROC) curve analysis and validation against external datasets. Notably, stratification based on the model demonstrated statistically significant correlations with patient survival outcomes. Furthermore, protein–protein interaction network analyses unveiled several pathways pertinent to tumor biology and immune responses. Intriguingly, the low-risk cohort exhibited enhanced immune infiltration, with the density of Th1 cell infiltration showing a positive correlation with increased patient risk, thereby highlighting its potential as a prognostic biomarker. Differential gene clustering analysis further underscored the critical role of ER stress models in prognostic predictions. Finally, our study identifies the IL4 signaling pathway is significantly associated with a good prognosis (*p* < 0.01), and may play a potential protective role for osteosarcoma, observed at the single-cell level by modulating macrophage polarization. The cause and effect relationship needs to be confirmed.

**Conclusion:**

Our findings suggest that evaluating endoplasmic reticulum stress levels and associated models in osteosarcoma patients could inform clinical interventions and enhance patient outcomes.

## Graphical abstract

Schematic diagram of the prognostic framework for dynamic integration of endoplasmic reticulum stress in osteosarcoma.
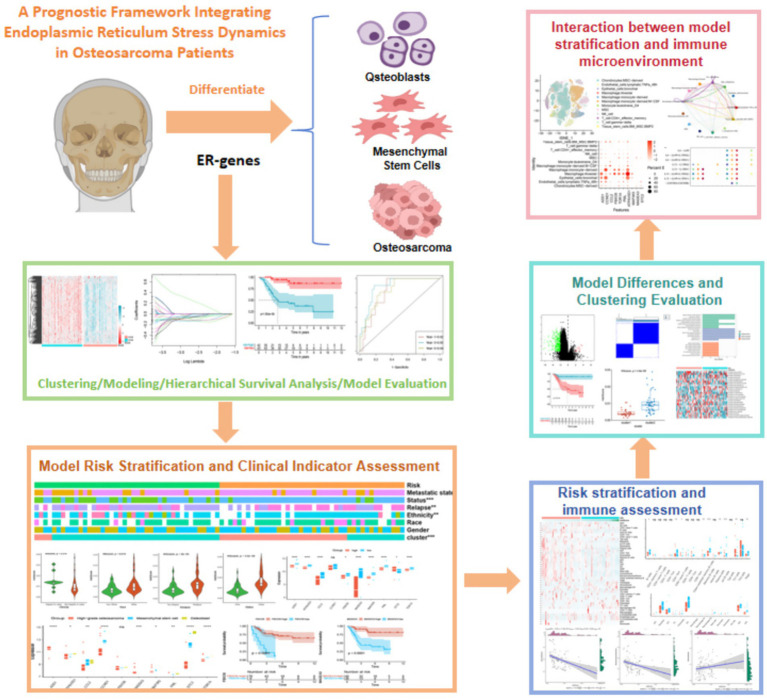

## 1 Introduction

Osteosarcoma mainly originates at the end of the long bone such as the outer part of the femur, the proximal tibia, which accounts for about 5% of solid tumors in children, has a high metastatic potential, and the incidence of lung metastasis reaches 40%, which is one of the leading causes of cancer-related death in adolescents (1). This alarming statistic has galvanized extensive research efforts aimed at enhancing therapeutic strategies and improving patient prognoses (2, 3). Bioinformatics has emerged as a pivotal tool in these investigations, facilitating the analysis of miRNA, lncRNA, and circular RNA in osteosarcoma, alongside genome-wide analyses to pinpoint viable biomarkers for treatment (4–7). Additionally, investigations into immune cell infiltration within the osteosarcomatous microenvironment have aimed to elucidate their influence on disease outcomes and to delineate potential avenues for immune modulation (8, 9). Accumulating evidence indicates that factors such as cellular apoptosis, the tumor microenvironment (TME), competitive endogenous RNA, and endoplasmic reticulum stress (ER stress) play integral roles in the pathogenesis and progression of osteosarcoma. However, the intricate relationship between ER stress and osteosarcoma remains insufficiently elucidated.

In the complex landscape of cancer evolution, the tumor microenvironment (TME) the TME exerts multifaceted metabolic stressors on infiltrating immune cells, encompassing acidosis, hypoxia, nutrient deprivation, and ER stress (10, 11). The endoplasmic reticulum (ER) is indispensable for maintaining cellular homeostasis and functionality, overseeing a myriad of fundamental processes. The onset of the ER stress response is instigated by the misfolding and accumulation of proteins in the ER lumen, coupled with the dysregulation of calcium balance. This cascade activates mechanisms such as the unfolded protein response, the ER overload response, and the caspase-12 mediated apoptosis pathway, which ultimately impede tumor progression by modulating the behavior of myeloid cells within the TME (12, 13). Moreover, the intrinsic ER stress response of malignant cells has been shown to significantly affect tumor evolution by influencing the functionality of concomitant immune cell populations (14). Notably, XBP1, a pivotal regulator of ER stress, has been associated with lipid metabolism in dendritic cells (DCs), subsequently inhibiting anti-tumor immune responses in T cells while facilitating tumor advancement (15). Recent breakthroughs in single-cell RNA sequencing technologies have provided an enriched understanding of the heterogeneous and phenotypically diverse cell populations that populate the immune microenvironment (16).

Despite the recognized significance of ER stress in the framework of cancer pathogenesis, few studies have comprehensively explored its implications in osteosarcoma through bioinformatics-driven analysis. Consequently, the establishment of a robust model and the identification of predictive prognostic biomarkers related to ER stress in osteosarcoma emerge as critical endeavors, potentially offering reliable therapeutic targets to enhance patient outcomes.

In this study, we propose a novel model of ER stress and identify candidate genes associated with this pathway in the context of osteosarcoma. We systematically investigate the role of ER stress in the disease, culminating in the development of an innovative prognostic model that strives to uncover biomarkers for improved prognostication through bioinformatics. Additionally, our analysis incorporates aspects of immune cell infiltration. Intriguingly, at the single-cell level, the IL4 signaling pathway has been elucidated as conferring a favorable prognosis for osteosarcoma patients through both the developmental and progressive phases of the disease. This finding offers new insights into potential clinical interventions for patients grappling with osteosarcoma.

## 2 Methods

### 2.1 Data collection and endoplasmic reticulum-related gene acquisition

In order to investigate the biological characteristics of osteosarcoma, we conducted data collection and endoplasmic reticulum (ER)-related gene acquisition. We collected multiple transcriptome data related to osteosarcoma from various sources. Specifically, we enrolled 88 patients with osteosarcoma from the therapeutically applicable research to generate effective treatments (TARGET) database (https://ocg.cancer.gov/programs/target) and screened two osteosarcoma transcriptome data acquisition numbers from the GEO database (https://www.ncbi.nlm.nih.gov/geo/) as GSE33383 and GSE21257. To examine the potential biological functions and characteristics of ER stress in patients with osteosarcoma, we collected 256 previously published ER stress-related genes (17). We conducted a thorough investigation of their potential associations with osteosarcoma.

### 2.2 Principal component analysis

The implementation of principal component analysis on ER stress genes in osteosarcoma patients was executed utilizing the “factoExtra” package in conjunction with R 4.1.0 software.

### 2.3 Consistent clustering

To explore the variance in expression levels of ER stress genes in patients with osteosarcoma, we initially standardized Log2 (Gene Expression +1) using the transcriptome data of these patients. Subsequently, we conducted a consensus clustering of ER genes, utilizing the unsupervised clustering algorithm incorporated within R 4.1.0 software’s “ConsensusClusterPlus” package (18).

### 2.4 Construction of ER stress prognosis model

Fifty genes that have been found to be associated with the prognosis of patients suffering from osteosarcoma were carefully screened using univariate Cox regression. Subsequently, a comprehensive analysis was undertaken utilizing the least absolute shrinkage and selection operator (LASSO) algorithm to identify ten ER stress-related genes that were then used to establish a prognostic model for the disease. These ten genes comprise ADD1, CCL2, CCND1, STC2, FBXO6, TOR1A, PML, ATP6V0D1, MAP3K5, and MAGEA3. The ER stress-related scores were calculated through the utilization of the following formula: risk score = ∑ I [Coefficient(mRNAi) × Expression(mRNAi)]. Based on the computed scores, patients were categorized into either high-risk or low-risk groups. The survival curve was established with the aid of the “survminer” of R 4.1.0 package while the time-dependent receiver operating characteristic curve (ROC) was created utilizing the “timeROC” package of R 4.1.0 software. The methodology employed to verify the set was identical to the one described above.

### 2.5 Protein network interaction and pathway enrichment analysis

Our screening process was conducted with exceptional rigor to identify molecules that exhibited a strong correlation (>0.7) with the model compounds. The resulting protein network interaction diagram was meticulously constructed using the software, Cytoscape 3.6.1. Furthermore, the prognostic potential of the identified proteins was comprehensively evaluated by leveraging sophisticated computational techniques, such as GO and KEGG pathway enrichment analysis, which were executed using the “clusterProfiler” package in R 4.1.0.

### 2.6 Immunity correlation analysis

We employed rigorous data standardization methods to analyze transcriptome data from patients with osteosarcoma, with the objective of elucidating immune cell infiltration patterns. To achieve this, we utilized the “Xcell” package in R 4.1.0 software, which enabled the enrichment of 64 cells and matrix, immune, and microenvironment scores, including an array of immune cells (19). Furthermore, we conducted a comprehensive stratification analysis of ER stress.

### 2.7 Differential analysis and gene set enrichment analysis (GSEA)

We conducted an differential analysis of ER stress by comparing high and low-risk groups, and by setting stringent parameters of |logFC| > 2 and *p* < 0.05, we obtained 302 ER stratified prognostic genes that were crucial for determining the prognosis and survival of patients with osteosarcoma. Moreover, through consistent clustering methods, we were able to identify the significant pathways that these genes were involved in. To further elucidate their functions, we performed pathway enrichment analysis using the “clusterProfiler” package in R 4.1.0, which allowed us to identify the GO pathways that were enriched. Finally, we annotated the 302 genes using gene set variation analysis (GSVA), and through H.all.v7.4. Symbols.gmt, we obtained 25 hallmark pathways that were significantly enriched.

### 2.8 Single cell data analysis

Single-cell data were procured from the GEO database, yielding a total of 11 osteosarcoma samples with the GSE152048. We meticulously conducted preprocessing measures (nUMI > = 500) & (nGene > = 250) & (log10GenesPerUMI > 0.80) & (mitoRatio < 0.20), resulting in the screening of 128,259 cells. To eliminate batch effects, we standardized the 11 samples by “harmony” package of R 4.1.0 (Supplementary Figures S1A,B). Through T-Distributed Stochastic Neighbor Embedding (TSNE) dimension reduction, these cells were segregated into 45 subpopulations with a resolution of 0.8(Supplementary Figure S1C). Cell cycle states of 11 samples were calculated by CellCycleScoring function (Supplementary Figure S1D). We employed the R 4.1.0 package “SingleR” and consulted the “HumanPrimaryCellAtlasData” database to divide these subpopulations into 12 distinct cell types, namely, Endothelial cells:lymphatic: TNFa_48h, Epithelial cells:bronchial, Macrophage: Alveolar, Macrophage:monocyte−derived, Macrophage:monocyte−derived: M − CSF, Monocyte:leukotriene_D4, MSC, NK cell, T cell: CD4 + _effector_memory, T cell: gamma−delta, Tissue stem cells: BM MSC: BMP2, and Chondrocytes: MSC − derived. We further segregated chondrocytes into high malignant and low malignant groups based on the expression of 9 model genes, using the AddModuleScore function. Finally, we constructed a cell communication network between osteosarcoma cells and other cells using the R 4.1.0 package “CellChat.”

### 2.9 Other statistical methods

We employed rigorous statistical methods to analyze our data, including the wilcox test for two-group comparisons and the kruskal-wallis’s test for comparisons between three or more groups. Additionally, we utilized the log-rank test to assess the statistical differences in overall survival between groups, and the time-dependent ROC to validate the efficacy of our model. To determine whether stratification of ER stress was associated with other clinical factors, we utilized the chi-square test. Fisher’s exact probability test is used to examine whether there is a significant association between classifications in multiple groups. Furthermore, correlation analysis was conducted using the pearson correlation test. All *p* values were bilateral, with statistical significance defined as *p* < 0.05.

## 3 Results

### 3.1 Differential gene analysis of ER stress-related genes in osteosarcoma, osteoblasts, and mesenchymal stem cells

**Figure 1:**
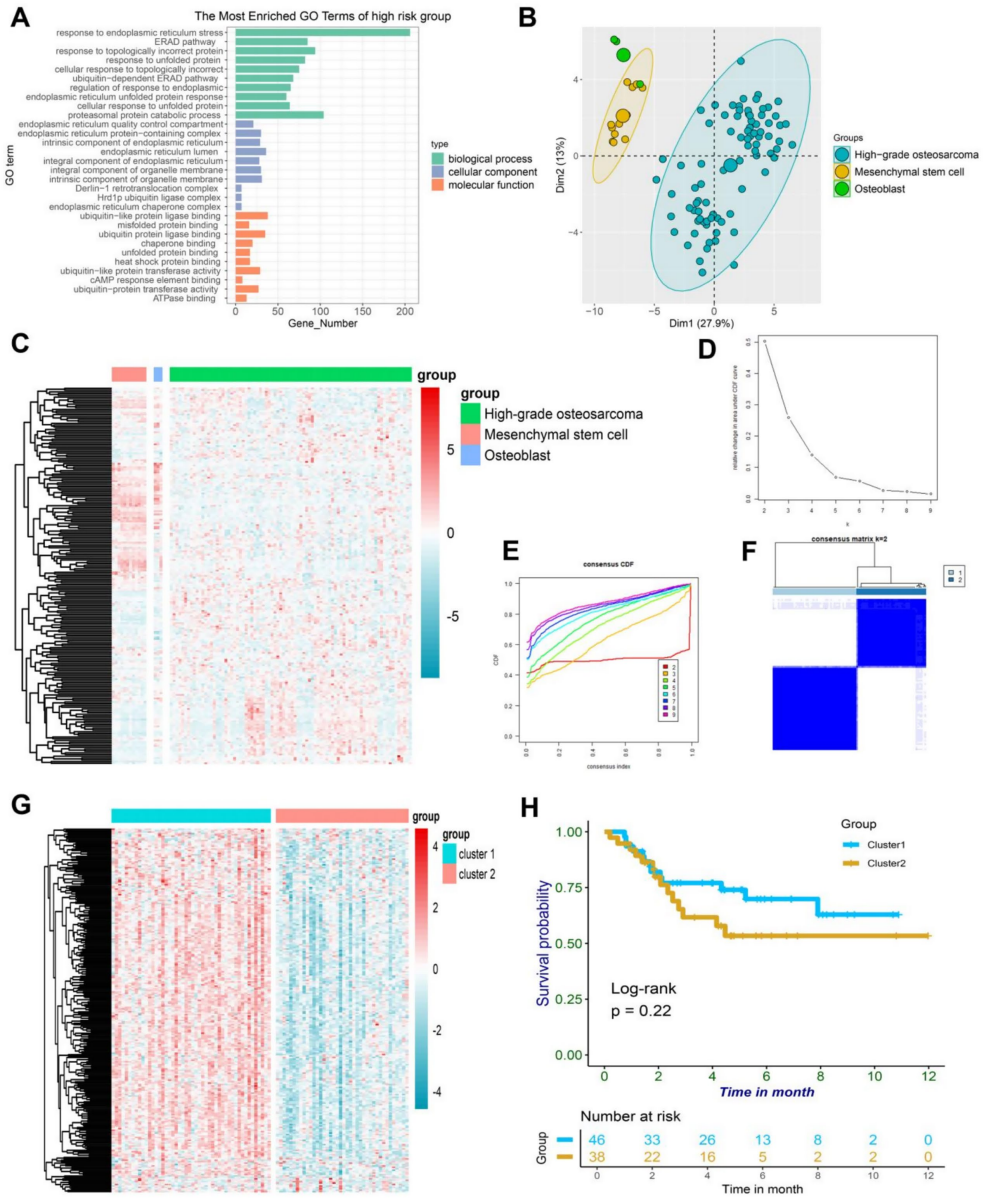
**(A)** A bar plot depicting the results of GO enrichment analysis on a collection of 256 ER-associated genes revealed their active involvement in ER-associated pathways. **(B)** The PCA plot of ER-associated genes revealed distinct variations in osteoblasts, mesenchymal stem cells, and high-grade osteosarcoma. **(C)** The heatmap demonstrated differential expression patterns of ER-associated genes in osteoblasts, mesenchymal stem cells, and high-grade osteosarcoma. **(D)** The delta area plot from consensus clustering analysis of ER-associated genes highlighted their significance in osteosarcoma. **(E)** The consensus consistent cumulative distribution function (CDF) plot from consensus clustering analysis of ER-associated genes provided further insight into their relevance in osteosarcoma. **(F)** The cluster heatmap resulting from consensus clustering analysis of ER-associated genes unveiled distinct patterns in osteosarcoma. **(G)** The heatmap displayed differential expression of ER-associated genes between cluster1 and cluster2 in osteosarcoma, with higher expression in cluster1. **(H)** The Kaplan–Meier curve illustrated the survival differences between cluster1 and cluster2 in osteosarcoma.

It has been unequivocally established that ER stress is a critical regulator of various precancerous characteristics and immune cell reprogramming, thereby playing a pivotal role in cancer progression. Delving deeper into this phenomenon, we conducted an analysis of 256 ER stress genes to determine their potential biological functions in osteosarcoma patients (17). Gene ontology enrichment analysis revealed that these genes primarily function in the ER stress pathway and other protein folding pathways, which are crucial for comprehending the impact of ER stress on cancer (Figure 1A). Principal component analysis of osteosarcoma data in GSE33383 indicated that ER stress-related genes could be segregated into three distinct subgroups in osteoblasts, mesenchymal stem cells, and osteosarcoma (Figure 1B), highlighting the heterogeneous nature of ER stress in osteosarcoma patients. This heterogeneity was further demonstrated through a heatmap depicting the differential expression of ER stress genes in osteosarcoma, osteoblasts, and mesenchymal stem cells (Figure 1C). Through the utilization of the TARGET database, we were able to partition the ER stress related genes in osteosarcoma patients into distinct clusters via the implementation of consistent clustering techniques. Based on our theoretical framework, it was deemed appropriate to separate the osteosarcoma patients into two distinct clusters, namely cluster 1 and cluster 2, as delineated by the CDF and CDF curve (Figures 1D–F). Upon further examination of the heatmap in Figure 1G, it became apparent that the expression of ER stress genes in cluster 1 was significantly higher than in cluster 2. However, the survival analysis indicated no significant difference between cluster 1 and cluster 2 (*p* = 0.22), signifying that mere classification of osteosarcoma patients through consistent clustering was insufficient to accurately predict prognosis (Figure 1H). Thus, further analysis of the patients’ endoplasmic reticulum stress genes was required. In summary, our findings underscore the pivotal role that endoplasmic reticulum stress plays in osteosarcoma, and highlight the need for a more comprehensive investigation into the prognosis of patients afflicted with this disease.

### 3.2 Construction of ER stress prognostic model

**Figure 2:**
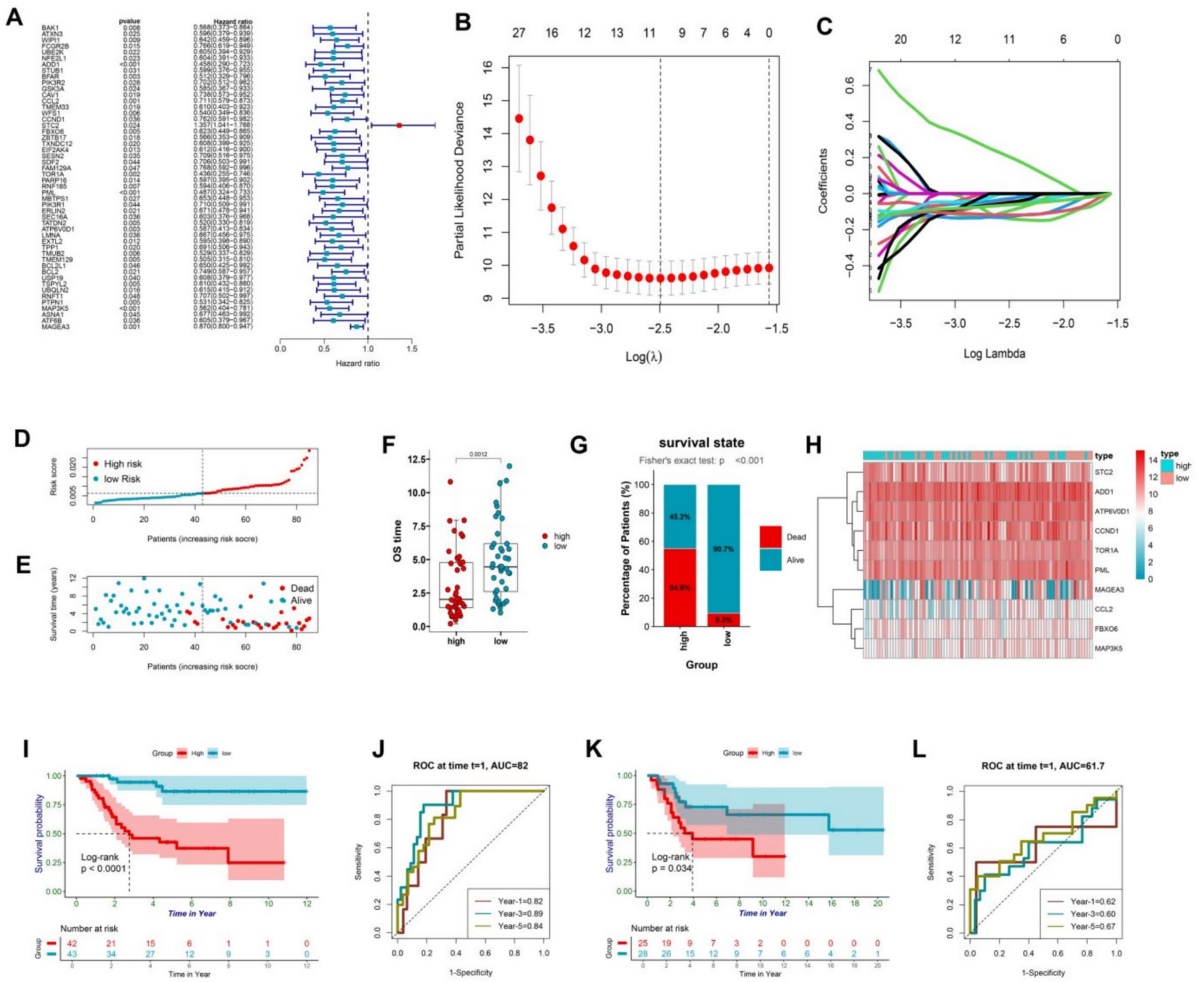
**(A)** ER-associated genes with prognostic significance were identified based on univariate Cox proportional hazards regression with statistical significance (*p* < 0.05). **(B,C)** Minimum criteria were calculated using the LASSO regression method for feature selection. **(D)** The patients were stratified into high and low-risk groups based on the risk scores generated by the protective model. **(E)** The dot plot demonstrated a correlation between increasing risk scores and patient mortality, as well as shorter survival time. **(F)** The box plot shows the difference in survival duration between the high-risk and low-risk groups. **(G)** The percentage chart shows the proportion of the number of survivors and deaths between the high-risk and low-risk groups. **(H)** The heatmap visualized the differential expression of protective model genes between the high and low-risk groups. **(I)** The Kaplan–Meier curve analysis of the training data from TARGET revealed significant differences in survival outcomes between the high and low-risk groups (*p* < 0.05). **(J)** The plot presented the 1-, 3-, and 5-year ROC curves of the risk model for the training data from TARGET, showing AUC values of 0.82, 0.89, and 0.84, respectively. **(K)** The Kaplan–Meier curve analysis of the test data from GSE21257 confirmed the reliability of the risk model, demonstrating statistically significant differences in survival outcomes between the high and low-risk groups (*p* < 0.05). **(L)** The time-dependent ROC curve for the test data from GSE21257 further validated the predictive accuracy of the model, with AUC values of 0.62, 0.60, and 0.67 for 1, 3, and 5 years, respectively, indicating that the model performs well over time.

In order to investigate the correlation between ER stress-related genes and the clinical outcome of osteosarcoma, a comprehensive analysis was conducted on 256 genes associated with endoplasmic reticulum stress, which were extracted and filtered from the osteosarcoma data in the TARGET public database. Subsequently, 50 genes that were significantly associated with the prognosis of osteosarcoma in children were selected for further investigation using cox regression analysis. Among these genes, STC2 was identified as a critical risk factor with a risk value exceeding 1, whereas the remaining 49 genes served as protective factors with risks below 1 (Figure 2A). To delve deeper into these genes, 10 ER stress-related molecules were identified using Lasso regression analysis, which included ADD1, CCL2, CCND1, STC2, FBXO6, TOR1A, PML, ATP6V0D1, MAP3K5, and MAGEA3. These molecules were used to construct a prognosis model (Figures 2B,C). The ER stress-osteosarcoma prognosis risk models were established as follows: ERS = ADD1*(−0.14) + CCL2*(−0.05) + CCND1*(−0.03) + STC*(0.20) + FBXO6*(−0.03) + TOR1A*(−0.09) + PML*(−0.13) + ATP6V0D1*(−0.05) + MAP3K5*(−0.12) + MAGEA3*(−0.06). Based on the gene expression levels of each patient, the risk value of the patient was calculated to evaluate their prognosis, and the patients were classified into high-risk and low-risk groups accordingly. As the risk scores of in patients increased, their prognosis grew increasingly dire, with shorter survival time (Figures 2D,E). The results revealed that the low-risk group demonstrated markedly extended survival durations and a greater count of surviving individuals compared to the high-risk group (Figures 2F,G). This was demonstrated by a heatmap illustrating gene expression in the two patient groups (Figure 2H), as well as by a survival curve, which revealed that patients in the low-risk group had significantly (*p* < 0.001) better survival rates than those in the high-risk group (Figure 2I). To evaluate the model’s efficacy, we generated a time-dependent ROC for osteosarcoma patients, which indicated good predictive value with areas under the ROC curve of 0.82, 0.89, and 0.84 in 1, 3, and 5 years, respectively (Figure 2J). Subsequently, we assessed the model’s stability by analyzing another sequencing dataset of osteosarcoma patients (GSE21257), which confirmed that the model was reliable in both high and low-risk groups, as demonstrated by statistically significant survival curves in both groups (*p* < 0.05) (Figure 2K). The time-dependent ROC curve also revealed favorable results, with areas under the ROC curve reaching 0.62, 0.60, and 0.67 after 1, 3, and 5 years, respectively (Figure 2L). Altogether, this model effectively captures the prognostic characteristics of osteosarcoma patients, and may be used to guide clinical decision-making and improve patient outcomes.

### 3.3 Model stratification for the clinical patient characteristics

**Figure 3:**
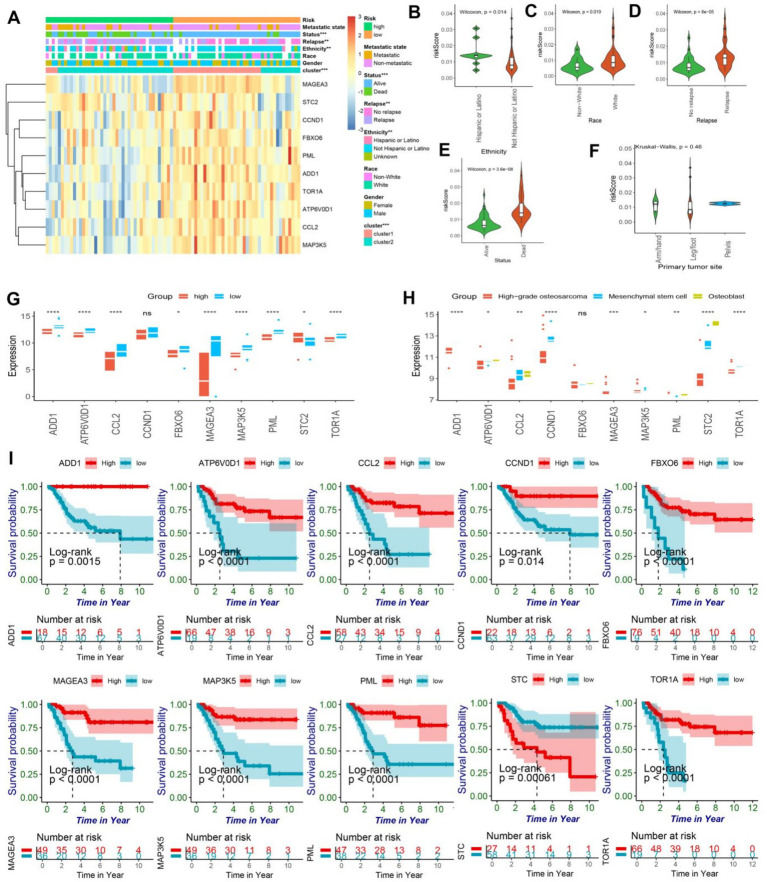
**(A)** The heatmap illustrated the expression patterns of prognostic genes in both high and low-risk groups of osteosarcomas, while the abscissa was additionally annotated with metastatic state, status, relapse, ethnicity, race, gender, ER-clusters, and risk group. Statistical significance was determined using the chi-square test (*<0.05; ** < 0.01; *** < 0.001). **(B–E)** The violin plot exhibited the disparity in risk scores among osteosarcoma patients based on their ethnicity, race, relapse, and status. **(F)** The violin plot revealed no significant difference in risk scores for primary tumor sites, including arm/hand, leg/foot, and pelvis. **(G)** The box plot showed the expressing differences in the 10 model genes between the high and low risk groups (ns, no significance; *<0.05; ** < 0.01; *** < 0.001;**** < 0.0001). **(H)** The box plot showed the differences in expression of 10 model genes in osteoblasts, mesenchymal stem cells, and high-grade osteosarcoma (ns, no significance; *<0.05; ** < 0.01; *** < 0.001;**** < 0.0001). **(I)** The Kaplan–Meier curve of the protective model’s prognostic genes, encompassing ADD1, CCL2, CCND1, STC2, FBXO6, TOR1A, PML, ATP6V0D1, MAP3K5, and MAGEA3, demonstrated significant difference between high and low-risk groups (*p* < 0.05).

The survival analysis revealed that the endoplasmic reticulum stress model demonstrated a remarkable predictive capacity in patients afflicted with osteosarcoma. Evidently, the stratification of the model was significantly associated with the clinical characteristics of osteosarcoma patients as demonstrated by the chi-square test (Figure 3A). Ten genes were filtered out from the model, and intriguingly, STC2 was highly expressed in the high-risk group, while the other nine genes were highly expressed in the low-risk group. Moreover, the stratification of the model was remarkably linked with cancer recurrence, survival status, human race, and ER cluster grouping.

Notably, Hispanic or Latino patients exhibited a higher prognosis-risk score, while white patients had higher risk scores (Figures 3B,C). Furthermore, in concurrence with our previous analyses, higher risk scores were observed in recurrent and deceased patients, thereby affirming the model’s prognostic value to a certain degree (Figures 3D,E). However, the location of osteosarcoma lesions did not significantly impact patients’ prognosis (Figure 3F). It is noteworthy that the expression levels of ADD1, CCL2, CCND1, FBXO6, TOR1A, PML, ATP6V0D1, MAP3K5, and MAGEA3 were found to be significantly lower in the high-risk group while STC2 exhibited a considerably higher expression level in the same group, as evidenced by statistical analysis (Figure 3G). Furthermore, the expression patterns of 10 model genes demonstrated significant differences in the prognosis of osteosarcoma. Notably, high-grade osteosarcoma, osteoblasts, and mesenchymal stem cells displayed distinct expression profiles of model genes, except for FBXO6 and MAGEA3, the expression of the remaining eight genes was found to be downregulated in high-grade osteosarcoma (Figure 3H). The single gene prognostic analysis revealed that patients with high expression levels of ADD1, CCL2, CCND1, FBXO6, TOR1A, PML, ATP6V0D1, MAP3K5, and MAGEA3 exhibited a better prognosis, while those with low expression levels of these genes had a poorer prognosis (all the survival curves, *p* < 0.05) (Figure 3I). In contrast, osteosarcoma patients with high expression levels of STC2 demonstrated a worse prognosis (*p* < 0.05), which was consistent with our previous findings (Figure 3I). In conclusion, both the ER stress model and the single gene analysis are crucial in identifying biomarkers for osteosarcoma and providing important guidance in clinical decision making.

### 3.4 Protein–protein interaction (PPI) and pathway analysis of model genes

**Figure 4:**
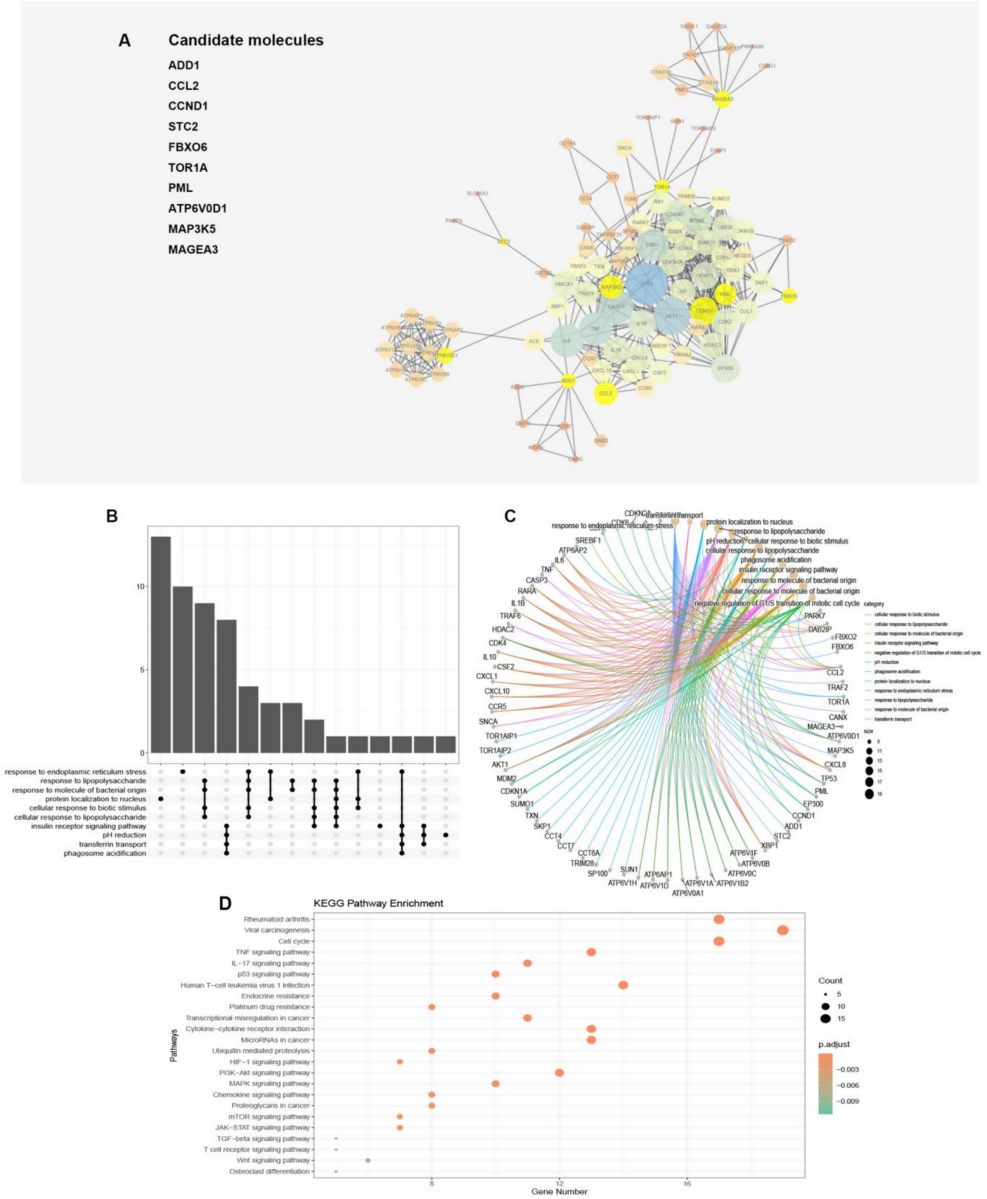
**(A)** The protein–protein network interactions of the 10 model genes were depicted in the visualization, with the 10 model genes highlighted in yellow. The size of the circles and thickness of the lines were indicative of the combined scores. **(B,C)** The plot illustrated that 89 proteins were linked to pathways via GO enrichment annotations. **(D)** The dot plot demonstrated the association between the 89 proteins and pathways through annotations of KEGG enrichment.

To investigate the potential function of the model genes’ protein, we utilized the STRING database to construct a protein interaction network (Figure 4A). Only those model gene proteins with a high interaction relationship (above 0.7) were selected to construct the potential function map, which highlighted the model genes’ pivotal position in the network. Impressively, a total of 89 proteins were found to have a relatively high interaction relationship with the model genes, providing a more comprehensive view of the model gene proteins’ network. Moreover, by performing GO analysis, we discovered that the proteins related to the model genes were involved in various pathways, such as cellular response to biotic stimulus, cellular response to lipopolysaccharide, cellular response to molecule of bacterial origin, negative regulation of G1/S transition of mitotic cell cycle, pH reduction, response to endoplasmic reticulum stress, transferrin transport, and response to lipopolysaccharide (Figures 4B,C). Moreover, through the implementation of KEGG pathway enrichment analysis, we have discovered that the genes in question are intricately involved in numerous pathways that are closely linked to cancer, such as viral carcinogenesis, transcriptional misregulation in cancer, and microRNAs in cancer, to name a few. Additionally, these genes are also associated with a number of immune-related pathways, including cytokine-cytokine receptor interaction and TGF-beta signaling pathway, as well as the differentiation of osteoclasts. Notably, these findings suggest that the molecules in our model exert a multifaceted impact on osteosarcoma, influencing critical processes such as endoplasmic reticulum stress, immunity, cancer, and osteoclast differentiation, among others (Figure 4D).

### 3.5 Relationship between model stratification and immune infiltration

**Figure 5:**
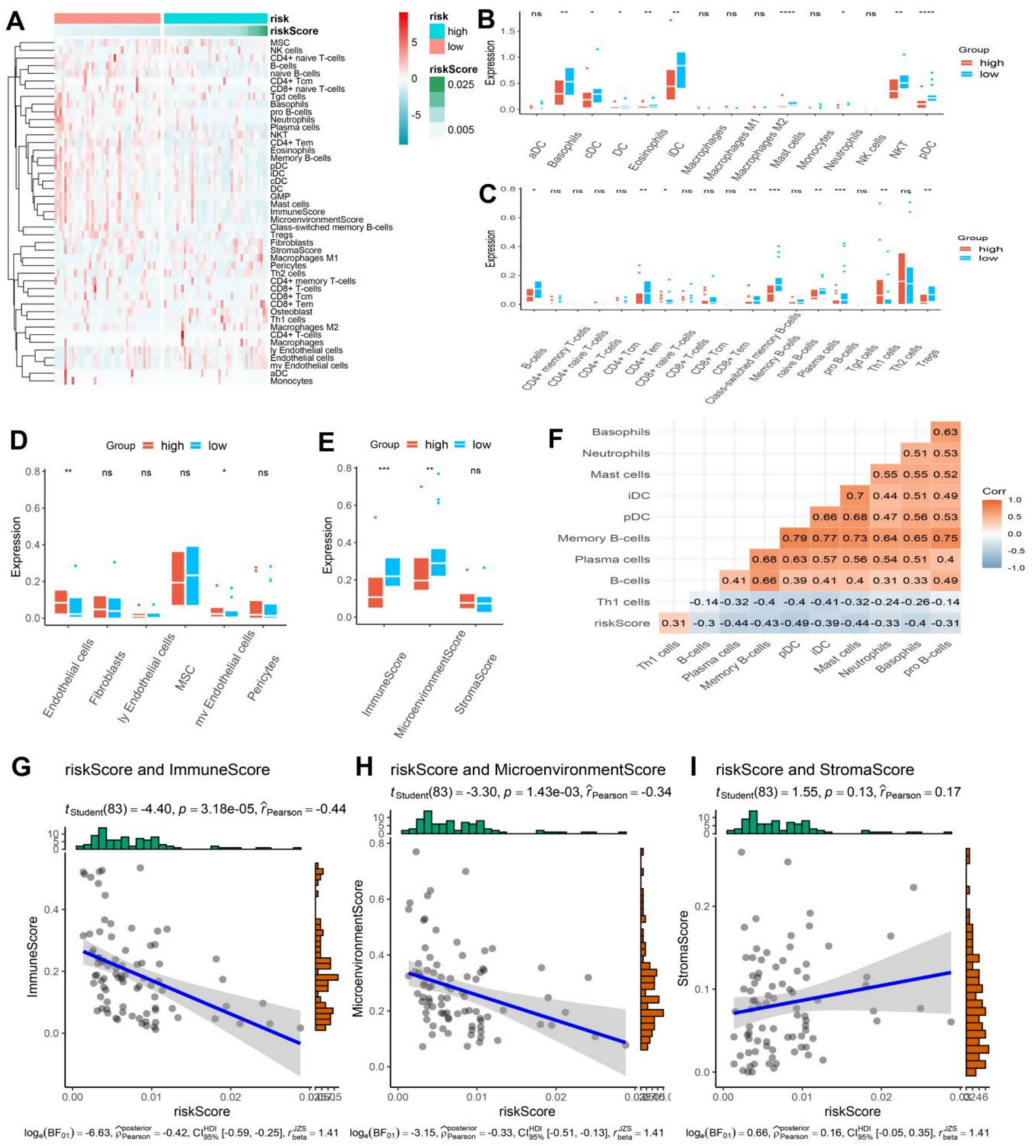
**(A)** The heatmap showcased the immune cell infiltration in osteosarcoma patients categorized into high and low-risk groups, displaying a higher infiltration of immune cells in the low-risk group. **(B–D)** The boxplot revealed distinct differences in innate, acquired immune cells, and other cells between the high and low-risk groups in osteosarcoma patients. **(E)** The boxplot exhibited contrasting immune, stromal, and microenvironment scores between the high and low-risk groups in osteosarcoma patients. **(F)** The correlation heatmap divulged a statistically significant association between the risk scores and other cells (|r| > 0.3). **(G–I)** The correlation plot demonstrated a significant association between the risk score and immune score and microenvironment score, while no correlation was observed with the stroma score.

Previous analysis has revealed a possible correlation between ER stress and immune function. To further investigate this connection, the xCell algorithm was utilized to calculate the level of immune cell infiltration in osteosarcoma patients. The resulting Heatmap provided a comprehensive summary of 64 cell types and a detailed overview of immunity, matrix, and the immune microenvironment (Figure 5A). Interestingly, certain cells were significantly overexpressed in the low-risk group. Within the innate immune cells, classical dendritic cells (cDC), immature dendritic cells (iDC), plasmacytoid dendritic cell (pDC), DC, Basophils, Eosinophils, Mast cells, Neutrophils, and natural killer T (NKT) cells were all found to be higher in the low-risk group (*p* < 0.05) (Figure 5B). Furthermore, within the adaptive immune cells, CD8 + naive T cells, CD4 + effector memory T cells (Tem), pro B cells, B cells, memory B cells, plasma cells, and regulatory cells (Tregs) were also more abundant in the low-risk group (*p* < 0.05), while the number of helper T cell (Th) 1 cells was higher in the high-risk group (Figure 5C). Studies have shown that in various types of cancer, including non-small cell lung cancer, rectal cancer, gastric cancer, and osteosarcoma, the deviation ratio of Th1/Th2 cells is positively correlated with the degree of tumor malignancy (20). Thus, the high expression of Th1 cells could potentially serve as an indicator of poor prognosis in osteosarcoma patients. Additionally, endothelial cells were found to be more infiltrated in the high-risk group (Figure 5D). The low-risk group of osteosarcoma patients exhibited a substantially elevated immune score and immune microenvironment score, in contrast to their high-risk counterparts, while no notable variance was detected in the matrix score (Figure 5E). To elaborate, the findings indicate that patients in the low-risk group demonstrated a more robust immune response, both innate and adaptive, which can serve as a valuable guide in the treatment of osteosarcoma patients. The study further conducted a correlation analysis between the model score and cells, specifically showcasing the heatmap of cell types exhibiting a correlation greater than 0.3. Interestingly, these cells demonstrated a negative correlation with other cells except for Th1 cells (Figure 5F). The model score was negatively correlated with the immune score and immune microenvironment score, and the correlation coefficients were statistically significant (−0.44 and −0.34, respectively) (Figures 5G,H). However, the correlation analysis between model scores and matrix scores did not exhibit statistical significance (Figure 5I). To summarize, the study underscores the close relationship between immune cells and the prognosis of osteosarcoma patients, with various immune cells exhibiting a negative correlation. Th1 cells also emerge as a promising biomarker for predicting the risk and prognosis of osteosarcoma patients.

### 3.6 Distinctive attributes of cohorts at high and low risk

**Figure 6:**
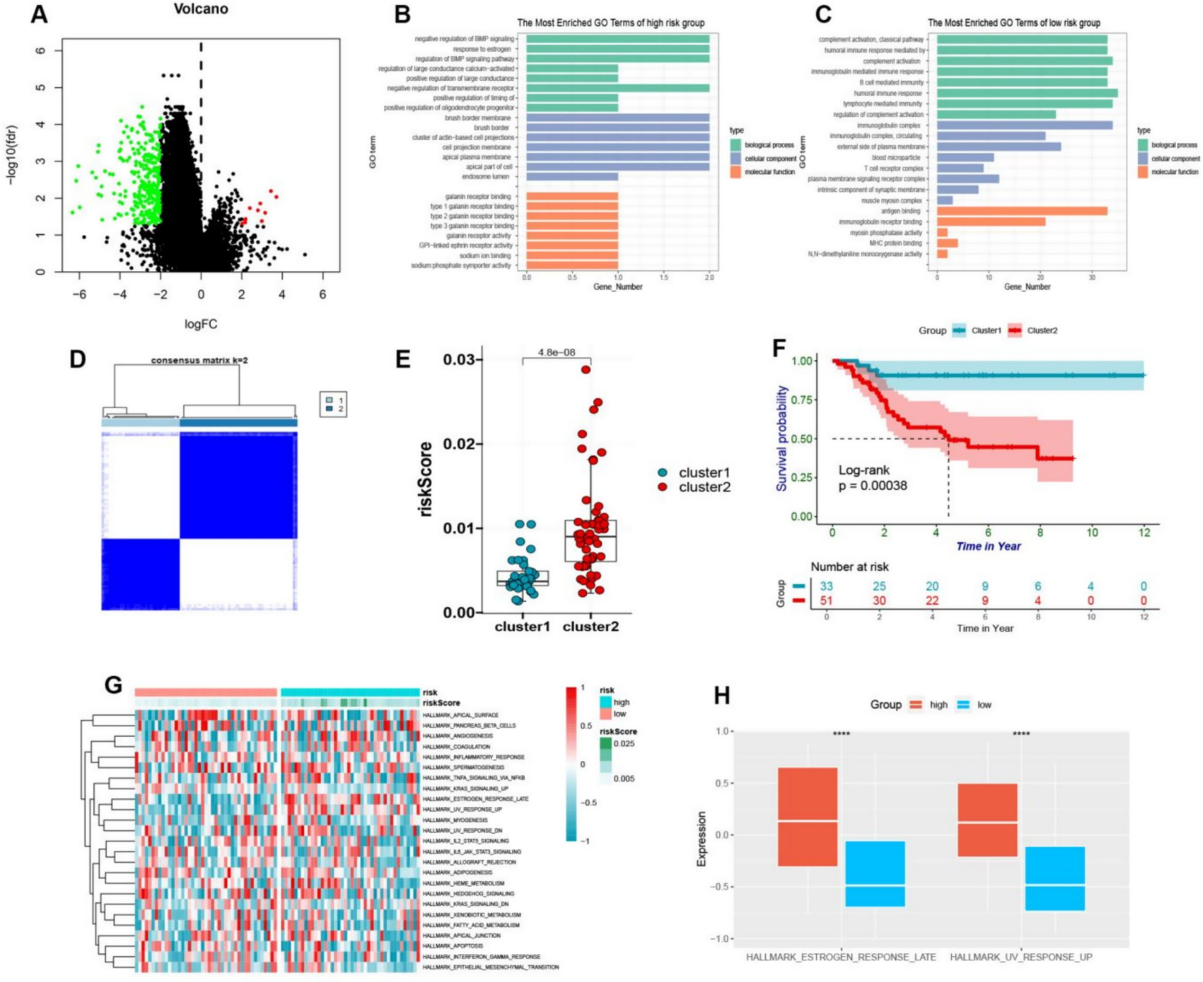
**(A)** The volcano plot visually depicted the differential expression of genes between high-risk and low-risk osteosarcoma groups, with upregulation illustrated by red and downregulation by green. **(B,C)** The GO enrichment analysis of upregulated genes in high-risk and low-risk groups was presented in a bar plot. **(D)** The cluster heatmap showed that different genes of high and low-risk was divided into two clusters. **(E)** By means of a boxplot, it was revealed that cluster B exhibited higher risk scores in comparison to cluster A. **(F)** The prognostic value of cluster A and cluster B was demonstrated in osteosarcoma patients through a Kaplan–Meier curve (*p* < 0.05). **(G)** The GSVA enrichment was used to generate a heatmap depicting pathway enrichment scores in high-risk and low-risk groups. **(H)** A boxplot was employed to display the higher enrichment levels of estrogen response late and UV response up in the high-risk group.

Based on our previous analysis, discernable disparities were detected in the clinical prognoses and immune spectra of osteosarcoma patients categorized into the high-risk and low-risk groups. To elucidate the specific biological effects, differential analysis was employed to isolate the differential genes between the high-risk and low-risk groups, from which 291 genes were observed to be down-regulated and 11 genes were up-regulated (Figure 6A). Further investigation via GO analysis revealed that the pathways implicated in the high-risk group encompassed the negative regulation of bone morphogenetic protein and estrogen response, large conductance calcium activates potassium channels and negatively regulates membrane receptors, thus influencing bone metabolism and the potential risk of osteoporosis (Figure 6B). In contrast, in the low-risk group, the pathways involved were complex activation, classical pathway, human immune response mediated, complex activation, immunoglobulin mediated immune response, B cell-mediated immunity, human immune response, lymphocyte-mediated immunity, and regulation of complex activation (Figure 6C). Hence, it can be deduced that patients categorized as low-risk exhibited a more robust immune response, which is in coherence with our previous analysis. Further, these differential genes were bifurcated into two clusters, i.e., cluster 1 and cluster 2, through cluster analysis (Figure 6D). Remarkably, the subtypes of these differential genes were found to be intricately linked with the survival and prognosis of osteosarcoma patients, with a statistically significant survival curve (*p* < 0.05), where patients belonging to cluster 1 showed a better prognosis (Figures 6E,F). By conducting GSVA analysis of these differential genes, we observed that these genes were enriched into 25 pathways. Additionally, the heatmap depicted the enrichment score of each patient (Figure 6G). Notably, we detected variations in estrogen response and ultraviolet response in the high-risk group, and the response in the high-risk group was more pronounced (Figure 6H).

### 3.7 The IL4 signaling pathway presents a promising protective mechanism for patients with osteosarcoma

**Figure 7:**
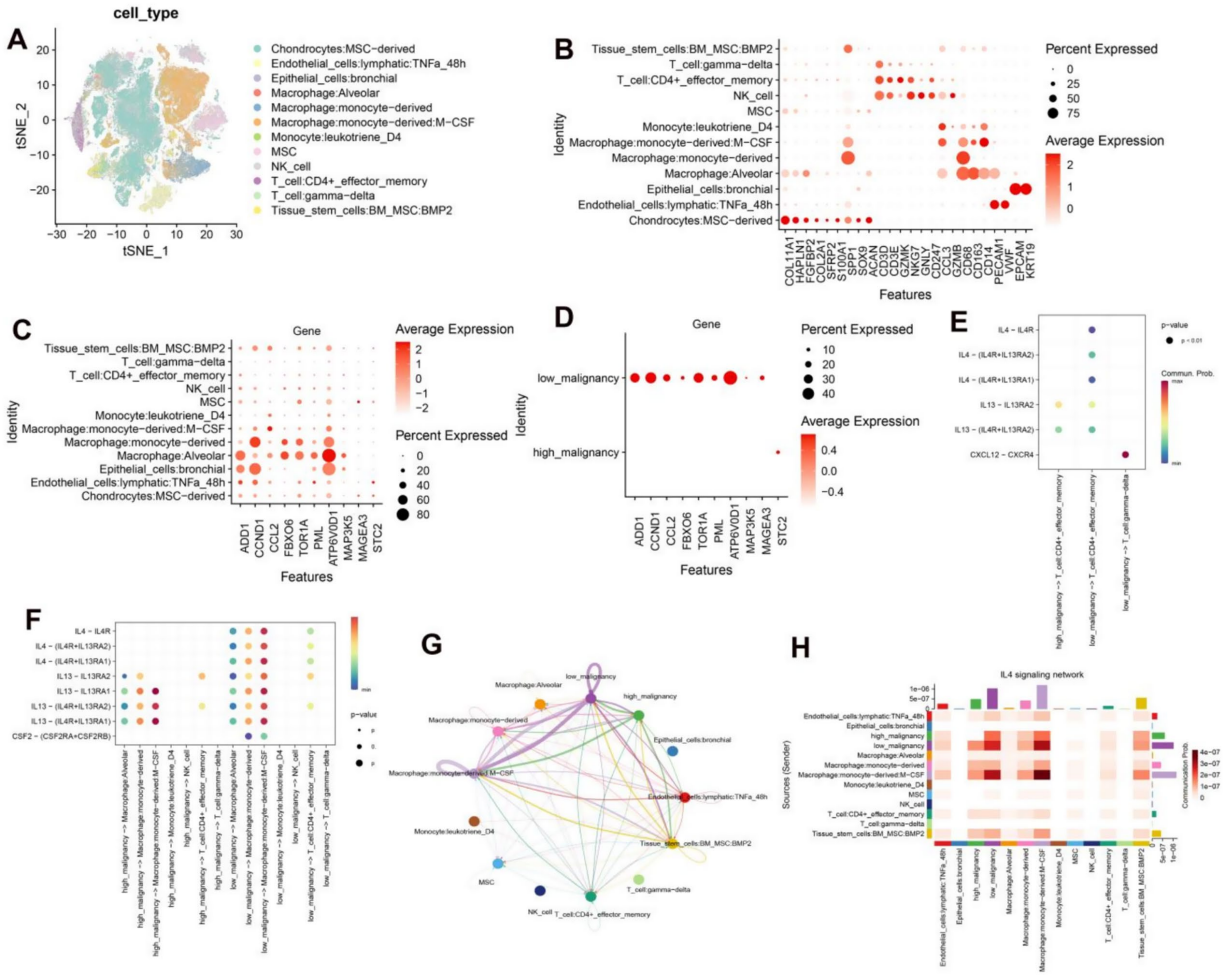
**(A)** Annotated TSNE plots depicting 12 distinct cell types. **(B)** Dot plots illustrate the average expression levels of marker genes across various cell types. **(C)** Dot plots display the average expression levels of 10 model genes across distinct cell types. **(D)** Dot plots demonstrate the average expression levels of 10 model genes in low and high malignant chondrocytes. **(E)** Dot plots depict the interaction of receptor and ligand pairs between low-malignant and high-malignant chondrocytes with T cells. **(F)** Dot plots showcase the interaction of receptor and ligand pairs between low-malignant and high-malignant chondrocytes with macrophages, monocytes, NK, and T cells. **(G)** Circular diagrams visually represent the interactions between low and high-malignant chondrocyte populations and other cell types. **(H)** Heatmaps effectively demonstrate the interactions between low and high-malignant chondrocyte populations and other cell types.

We delved into the pathway characteristics of the high and low risk cohorts at a single-cell resolution. We have conducted an analysis of single-cell transcriptome samples obtained from 11 osteosarcoma patients, which were further annotated with a total of 12 distinct cell types through the use of a comprehensive database (Figure 7A). Chondrocytes exhibited high expression of genes including COL11A1, HAPLN1, FGFBP2, COL2A1, SFRP2, S100A1, SPP1, SOX9 and ACAN. Endothelial cells demonstrated elevated levels of PECAM1 and VWF. Epithelial cells displayed marked expression of EPCAM and KRT19. Monocytes and macrophages were characterized by high levels of CD68, CD163 and CD14. T cells showed significant expression of CD3D and CD3E while NK cells exhibited heightened levels of NKG7, GNLY, CD247, CCL3, and GZMB (Figure 7B). The chosen model genes were found to be expressed across all 12 cell types, with a particularly high expression in macrophages (Figure 7C). We then categorized osteosarcoma cells into two groups based on the expression of these ten model genes: those with low malignancy showed significant expression of ADD1, CCL2, FBXO6, TOR1A, PML, ATP6V0D1, MAP3K5 and MAGEA3; while those with high malignancy exhibited elevated levels of STC2. This finding was consistent with our comprehensive analysis at the bulk transcriptome level (Figure 7D). Through ligand-receptor interaction analysis with T cells in both high and low malignant populations, it was observed that the receptor-ligand pairs of IL4 − IL4R, IL4 − (IL4R + IL13RA2), and IL4 − (IL4R + IL13RA1) were more highly expressed in the low malignant chondrocyte population (Figure 7E). Furthermore, the receptor ligand pairs of IL4 − IL4R, IL4 − (IL4R + IL13RA2), and IL4 − (IL4R + IL13RA1) were significantly elevated in macrophage interactions within the low-malignant chondrocyte population, while such properties were not observed in the high-malignant chondrocyte population (Figure 7F). The analysis of the IL4 signaling pathway revealed that the interaction within this pathway in low malignant subsets of macrophages significantly distinguished them from high malignant subsets, particularly Macrophages monocyte-derived: M-CSF (Figures 7G,H). The aforementioned findings suggest that IL4 signaling may have a protective effect in osteosarcoma patients and could serve as a potential biomarker with favorable prognostic characteristics.

## 4 Discussion

The incidence rate of osteosarcoma is alarmingly noted at 3.4 per million individuals, with its high-grade malignancy resulting in a dismal prognosis (21, 22). The etiology of this aggressive cancer is multifaceted, encompassing a variety of factors, including competitive endogenous RNA and endoplasmic reticulum (ER) stress, as well as associated cellular elements. Furthermore, osteosarcoma is characterized by striking genomic alterations and marked heterogeneity, which complicate treatment regimens (23, 24). Presently, standard therapeutic approaches—including surgical intervention and chemotherapy—fall short in improving patient outcomes (1, 25). This underscores an urgent need for the discovery of novel and effective biomarkers, as well as innovative treatment strategies tailored to this challenging malignancy.

In light of the emergence of publicly available databases, we undertook this study employing bioinformatics methodologies. While previous bioinformatics research on osteosarcoma has predominantly concentrated on miRNA, lncRNA, and circRNA, the role of ER stress biomarkers has received comparatively limited investigation and thus warrants thorough exploration. The endoplasmic reticulum plays a pivotal role in the critical processes of protein handling, modification, and folding, fundamentally influencing cellular function, survival, and apoptosis (26). Within the tumor microenvironment, ER homeostasis is frequently disrupted, with such ER stress emerging as a significant contributor to cancer progression (27). Several studies have elucidated the critical involvement of ER stress in osteosarcoma through diverse molecular pathways (28–30). Overactivation of ER stress in high-risk groups may inhibit the infiltration of disease-free cells through the PERK / eIF2a pathway (28), Moderate ER stress in the low-risk group promoted epidemic free response through activation of the NF-kB pathway (29). Recognizing the importance of ER stress in osteosarcoma progression, we delved into its functional implications and identified potential biomarkers.

Osteosarcoma is marked by the presence of osteoblastic mesenchymal stem cells, which give rise to osteoblasts, chondrocytes, and adipocytes (31, 32). Extensive research suggests that the pathogenesis of osteosarcoma may result from significant genetic alterations that impair the differentiation of osteoblasts from mesenchymal stem cells (33). Thus, our study highlights the potential roles of osteoblasts and mesenchymal stem cells in this disease.

In this investigation, we selected a cohort of 256 ER stress-related genes based on prior studies in gliomas, given the parallel functions of ER stress across various cancer types. Although 256 ER stress genes originated from glioma studies, their core functions (such as unfolded protein response and calcium homeostasis regulation) are highly conserved in nucleocytes. This study confirmed through the GO enrichment pathway that these genes present significantly different expression patterns in osteosarcoma than osteocytes, and that 10 genes (such as CCL2, STC2) after LASSO screening have been shown to be directly related to osteosarcomas progression (30, 34), demonstrating their applicability. A comprehensive analysis of the biological functions and gene ontology enrichment of these genes revealed a consistent association between ER stress and osteosarcoma. Moreover, considering the intricate relationship between osteosarcoma, osteoblasts, and mesenchymal stem cells, principal component analysis demonstrated clear evidence of heterogeneity among these cell types, corroborating previous findings on the characteristics of osteosarcoma.

To elucidate the role of ER stress genes in the progression of osteosarcoma, we stratified patients into two distinct groups, consistent with classical methodologies. However, our analysis returned no significant differences between the groups. Consequently, we developed a novel prognostic risk model for osteosarcoma using Cox and LASSO regression. This model effectively stratified patients into high-risk and low-risk categories. The robustness of our prognostic model was further validated through time-dependent ROC analysis and confirmation using the GSE21257 dataset, demonstrating its utility in predicting patient outcomes and offering valuable clinical insights. The low AUC value in the GSE21257 validation set (0.62 at 1 year) may be related to sample heterogeneity, but the significant difference in the K-M curve (*p* = 0.023) still confirms the clinical value of the model. In the future, model performance can be optimized by integrating methylation data.

Furthermore, we identified ten model genes (STC2, ADD1, CCL2, CCND1, FBXO6, TOR1A, PML, ATP6V0D1, MAP3K5, and MAGEA3) associated with prognosis, thus providing a roadmap for filtering potential biomarkers in osteosarcoma. STC2 as a risk factor (coefficient 0.20) in this model is consistent with the reports of “high expression of STC2 for poor prognosis of osteosarcoma” reported by Stefanidou et al. (35), further supporting its reliability as a core marker. At the same time, the differences between these studies (35, 36) and this model in analytical methods (such as multi-gene integration vs. single-gene analysis) were supplemented, highlighting the innovation of this study. To further investigate the interactions of these genes, we constructed a protein–protein interaction network, revealing that these model genes occupy central positions within a network of 89 interacting proteins.

It is widely recognized that the immune system plays a critical role in the progression of osteosarcoma (37, 38). Notably, our findings indicated elevated levels of various innate and adaptive immune cell types, including cDCs, iDCs, pDCs, DCs, basophils, eosinophils, mast cells, neutrophils, NKT cells, CD8 + naïve T cells, CD4 + Tem cells, pro-B cells, B cells, memory B cells, plasma cells, and Tregs. A prior study suggested that modulation of immune checkpoint pathways could represent a viable therapeutic strategy for osteosarcoma (34). Among the innate immune cells, NKT cells demonstrated cytotoxic effects against tumor cells, enhancing therapeutic efficacy and influencing the prognosis of osteosarcoma patients (39). Conversely, Th1 responses displayed a differential effect in our study, aligning with existing literature (40, 41). Collectively, Th1 cells may serve as potential biomarkers for osteosarcoma and the association of high Th1 cell infiltration with poor prognosis may be related to the expression of PD-L1 induced by the secretion of IFN-*γ* by Th1 cells in the osteosarcoma microenvironment. In this study, we explored the complex interplay between ER stress and osteosarcoma through the construction of an ER stress risk prognostic model, uncovering viable biomarkers that could improve prognostication in osteosarcoma patients.

We undertook a comprehensive annotation of single-cell types within the osteosarcoma database, revealing widespread expression of our ten model genes across all cell types. Additionally, our examination of chondrocytes at the single-cell level, categorized by varying malignant degrees, supported our bulk-level findings and highlighted the integral role of the IL4 signaling pathway in the immune response to osteosarcoma. Specifically, we identified key receptor-ligand interactions among IL4-IL4R, IL4-(IL4R + IL13RA2), and IL4-(IL4R + IL13RA1), mediating interactions between lower-malignancy chondrocyte subsets and T cells/macrophages. Notably, IL4 has been implicated in promoting the proliferation and migration of cancer cells across various malignancies (42–45). Conversely, the JAK2/STAT6 signaling pathway has been associated with inhibiting cancer cell proliferation, invasion, and metastasis (46). Consequently, the IL4 signaling pathway emerges as a promising biomarker for forecasting patient outcomes and guiding clinical management and early intervention in osteosarcoma. Future validation of the precise roles of the IL4 signaling pathway in osteosarcoma patients will necessitate extensive prospective clinical studies and fundamental experimental investigations.

This study has the following limitations: (1) small sample size (TARGET cohort *n* = 88) may affect the stability of the model, which requires large sample validation at multiple centers; (2) Depends on transcriptome data and does not involve protein level validation; (3) Public databases lack detailed clinical treatment information and cannot exclude interference of treatment regimens with prognosis; (4) The specific mechanism of IL4 pathway needs to be further elucidated *in vitro* functional experiments.

In conclusion, this study revealed the crucial role of endoplasmic reticulum stress in the progression of osteosarcoma, and established a prognostic risk model based on this mechanism, providing an innovative perspective for the discovery of new biomarkers and personalized treatment.

## Data Availability

The datasets presented in this study can be found in online repositories. The names of the repository/repositories and accession number(s) can be found below: the datasets generated during and/or analyzed during the current study are available in the public database TARGET and GEO database, for your convenience, the raw data associated with this study can be accessed through the following link “https://www.jianguoyun.com/p/DXQ_yIgQgcmVDRjw2-YFIAA.”

## Supplementary material

Supplementary Figure S1 **(A, B)** The tsne plots illustrate the distribution of 11 osteosarcoma samples before and after the removal of batch effects. **(B)** At a resolution of 0.8, the tsne plots depict the partitioning of osteosarcoma patients’ cells into 45 subgroups. **(D)** The scale plots provide a graphical representation of the relative distribution of cell cycles among the 11 osteosarcoma patients.
