# Supplementary figures and images for: A prognostic framework integrating endoplasmic reticulum stress dynamics reveals clinical stratification and differential prognostic attributes in osteosarcoma patients
Source: Front Med (Lausanne). 2025 Oct 1;12:1566387. doi: 10.3389/fmed.2025.1566387 (PMC12521199; doi:10.3389/fmed.2025.1566387)

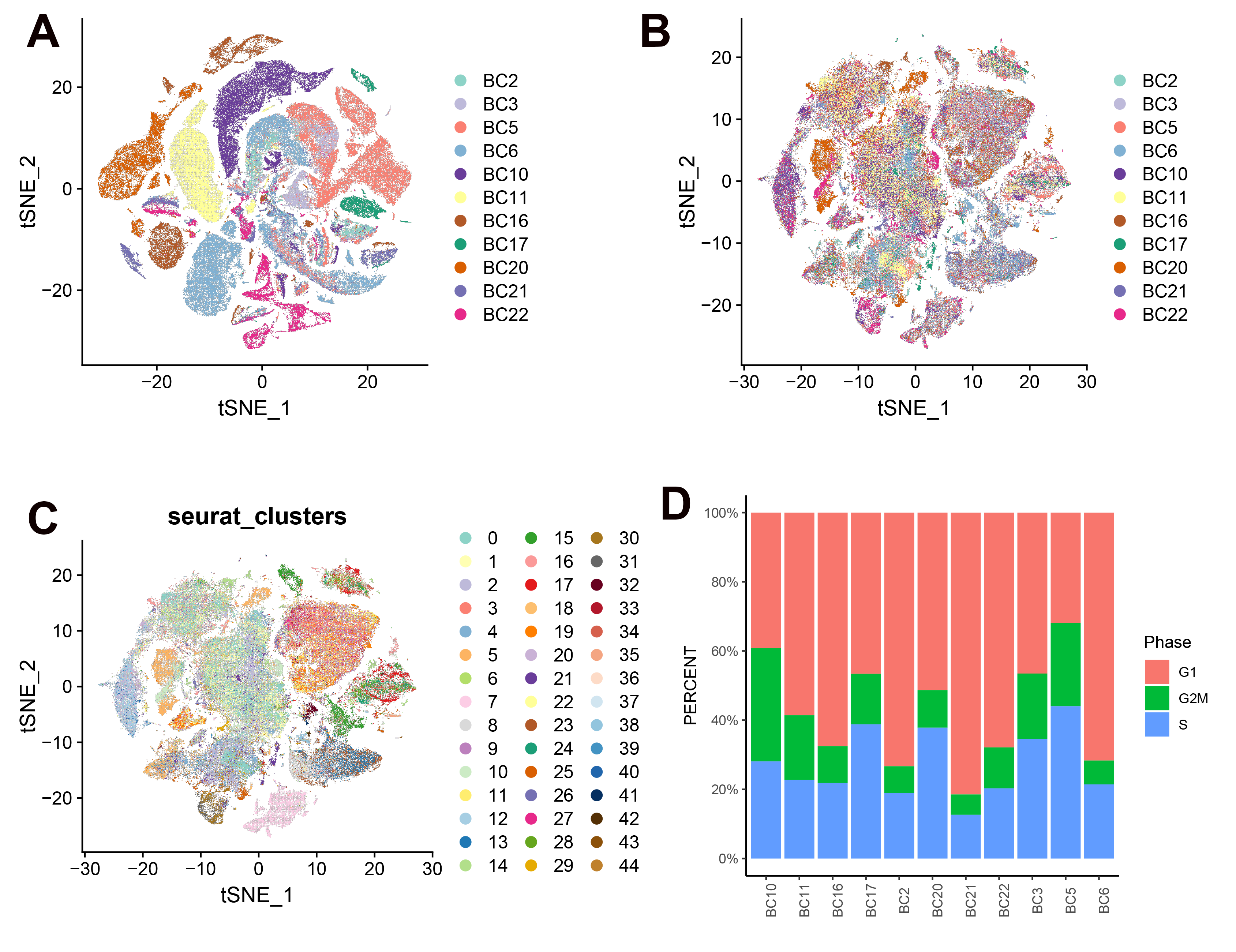

Supplement: Supplementary Figure S1 — (A, B) The tsne plots illustrate the distribution of 11 osteosarcoma samples before and after the removal of batch effects. (B) At a resolution of 0.8, the tsne plots depict the partitioning of osteosarcoma patients’ cells into 45 subgroups. (D) The scale plots provide a graphical representation of the relative distribution of cell cycles among the 11 osteosarcoma patients. [file Image_1.tif]
